# Evaluation of accuracies of genomic predictions for body conformation traits in Korean Holstein

**DOI:** 10.5713/ab.23.0237

**Published:** 2024-01-20

**Authors:** Md Azizul Haque, Mohammad Zahangir Alam, Asif Iqbal, Yun Mi Lee, Chang Gwon Dang, Jong Joo Kim

**Affiliations:** 1 Department of Biotechnology, Yeungnam University, Gyeongsan 38541, Korea; 2 Animal Breeding and Genetics Division, National Institute of Animal Science, Cheonan, 31000, Korea

**Keywords:** Accuracy, Body Conformation Traits, Genomic Prediction, Heritability, Korean Holstein

## Abstract

**Objective:**

This study aimed to assess the genetic parameters and accuracy of genomic predictions for twenty-four linear body conformation traits and overall conformation scores in Korean Holstein dairy cows.

**Methods:**

A dataset of 2,206 Korean Holsteins was collected, and genotyping was performed using the Illumina Bovine 50K single nucleotide polymorphism (SNP) chip. The traits investigated included body traits (stature, height at front end, chest width, body depth, angularity, body condition score, and locomotion), rump traits (rump angle, rump width, and loin strength), feet and leg traits (rear leg set, rear leg rear view, foot angle, heel depth, and bone quality), udder traits (udder depth, udder texture, udder support, fore udder attachment, front teat placement, front teat length, rear udder height, rear udder width, and rear teat placement), and overall conformation score. Accuracy of genomic predictions was assessed using the single-trait animal model genomic best linear unbiased prediction method implemented in the ASReml-SA v4.2 software.

**Results:**

Heritability estimates ranged from 0.10 to 0.50 for body traits, 0.21 to 0.35 for rump traits, 0.13 to 0.29 for feet and leg traits, and 0.05 to 0.46 for udder traits. Rump traits exhibited the highest average heritability (0.29), while feet and leg traits had the lowest estimates (0.21). Accuracy of genomic predictions varied among the twenty-four linear body conformation traits, ranging from 0.26 to 0.49. The heritability and prediction accuracy of genomic estimated breeding value (GEBV) for the overall conformation score were 0.45 and 0.46, respectively. The GEBVs for body conformation traits in Korean Holstein cows had low accuracy, falling below the 50% threshold.

**Conclusion:**

The limited response to selection for body conformation traits in Korean Holsteins may be attributed to both the low heritability of these traits and the lower accuracy estimates for GEBVs. Further research is needed to enhance the accuracy of GEBVs and improve the selection response for these traits.

## INTRODUCTION

Since the 1990s, body conformation traits have been widely used as indirect indicators of performance when judging dairy cattle in many countries [1]. In the dairy industry, the profitability of a cow is solely reliant on its milk production capacity. To ensure optimal performance, a cow should possess sound health, high fertility, superior feed efficiency, and a long and persistent productive life, which are essential for running a sustainable dairy production system. Breeding based on functional body shapes facilitated by body conformation traits holds significant importance for breeders due to their indirect association with milk production traits [2]. Furthermore, various reports suggest that body conformation traits exhibit genetic correlations with economically important traits such as calving ease, longevity, and lameness [3]. For instance, studies conducted on Iranian Holstein cows revealed that body conformation traits like stature and body depth exhibit genetic correlations with reproductive traits, including gestation length, calving interval, and days from calving to first insemination [4]. Although body conformation traits may not be direct economic traits for animal breeders, they are closely linked to other economically important traits such as the health [5], productivity [2], reproduction [6], profitability [7], and lifetime longevity of cattle [1]. Consequently, breeders consider different conformation traits such as udder depth, rump angle, rump width when selecting and judging dairy bulls, aiming to improve longevity and lifetime production [8].

The advent of high throughput single nucleotide polymorphism (SNP) chip technology in genomic selection (GS) has led to significant advancements in the genetic improvement of dairy cattle traits since 2009. Genomic selection has gained widespread usage and has become the preferred method over marker-assisted selection in many developed countries, primarily for dairy cattle, due to its advantages and rapid rates of genetic gains. Most dairy traits exhibit quantitative nature with complex genetic architecture, as they are controlled by multiple genes with small effects, and there is a strong influence of genotype-environment interaction. Therefore, the dairy cattle industry can greatly benefit from the implementation of GS programs, particularly for low heritable traits, traits that are difficult to measure, and sex-limited traits [9].

The success of GS depends on the accuracy of genomic prediction. Animals are selected based on their estimated breeding values (EBVs) rather than their true breeding values, which can be obtained in the early stages of life for traits that manifest later in life. Various statistical methodologies, including genomic best linear unbiased prediction (GBLUP) or ridge regression (RR-BLUP), as well as Bayesian methods, have been adapted to estimate genetic values based on genomic data. GEBV prediction using the GBLUP method relies on trait phenotypes and the genomic relationships among animals. Genomic relationships can be determined by utilizing high-density SNP markers, which exploit mendelian sampling effects and offer improved efficiency compared to traditional pedigree-based relationships [10].

Holsteins are the most popular dairy cattle breed worldwide due to their high milk production capacity. In Korea, this breed constitutes a significant portion of domestic milk production. The dairy cattle industry in Korea is focusing on developing sophisticated herd management programs that involve reducing the number of animals in herds to maximize production, profitability, and minimize methane emissions. However, the sudden culling of cows from the herd often poses a challenge. Therefore, cows with sound body conformation, high longevity, and persistent production are indispensable for maintaining a profitable and sustainable dairy production system. The Korea Animal Improvement Association (KAIA) has established general appearance and linear examination procedures for scoring dairy cows based on their body conformation. A total of 25 body conformation traits were screened by KAIA for estimation of genomic breeding values and their accuracy in this study. To the best of our knowledge, this is the first evaluation of the accuracy of genomic estimated breeding value (GEBV) for body conformation traits in Korean Holstein cattle. Hence, the present study was conducted to estimate the heritability, GEBVs, and their accuracies for body conformation traits in the Korean Holstein population. These findings will facilitate genetic improvement and selective breeding strategies, leading to enhanced productivity and performance within this breed.

## MATERIALS AND METHODS

### Animal management and phenotypes

The first parity phenotypic data were collected from 2,329 Holstein dairy cattle from Nonghyup livestock farms in Korea. The data were recorded from the year 2017 to 2018. The 25 traits were stature, height at front end, chest width, body depth, angularity, body condition score, locomotion, rump angle, rump width, and loin strength, rear leg set, rear leg rear view, foot angle, heel depth, bone quality, udder depth, udder texture, udder support, fore udder attachment, front teat placement, front teat length, rear udder height, rear udder width, rear teat placement, and overall conformation score. Phenotypic information was recorded for 25 body conformation scores based on the guidelines provided by the KAIA. The care and management of all animals used in this study were approved by the Animal Care and Use Committee of the National Institute of Animal Science (NIAS), Rural Development Administration (RDA), South Korea (Approval No. 2016-189). The body conformation traits consisted of 24 linear descriptive traits, which were scored on a scale from 1 to 9. One trait, overall conformation score, was measured using an index with values and scores ranging from 0 to 100. The measurements procedures for the linear body conformation traits are detailed in Table 1.

### Genotyping and quality control

Tail hair samples were collected from animals belonging to different commercial dairy farms in Korea to extract genomic DNA. All farmers provided permission to use their animal’s genetic material for this research. The genotyping procedure was carried out by the commercial genotyping service provider, DNA Link in Korea. A total of 2,329 Holstein dairy cows were genotyped using the Illumina Bovine SNP 50K v.3 Chip (Illumina Inc., San Diego, CA, USA), which contains a range of 53,218 to 54,609 embedded SNPs. To ensure data quality, all 29 autosomal SNPs were subjected to further quality control (QC) procedures. Several QC thresholds were applied to identify and remove poor-quality SNPs, selecting representative SNPs for subsequent GEBVs predictions. Single nucleotide polymorphisms were excluded from the analysis if they had a minor allele frequency of less than 5% (monomorphic), a SNP call rate below 90%, individuals with a genotyping call rate below 90%, or genotype frequencies that significantly deviated (p<10^–6^) from the Hardy-Weinberg Equilibrium. Additionally, an identity by state (IBS) test was conducted to identify any duplicate individuals or genotyping errors in the datasets. Pairs of individuals displaying a similarity rate greater than 99% were considered either identical animals or indicative of genotyping errors. The entire QC and IBS process was performed using the PLINK v1.9 toolset [14]. After the QC tests, 38,720 SNPs and 2,206 animals remained for further analysis.

### Statistical analysis

#### Estimation of variance components

The variance components and heritabilities were estimated using ASReml-SA v4.2 software [15]. The analysis was conducted using the genome-based single-trait animal model as follows:


y=Xb+Zu+e

where, y represents the vector of phenotypic records for n number of animals; b is the vector of the fixed effects, including birth year (14 levels), birth season (4 levels), test year (14 levels), test season (4 levels), and age at the recorded date (from 22 to 145 months) as a covariate; u is the vector of additive genetic effects of the individuals; X denotes the incidence matrices of b; Z is the incidence matrix of u and e is the vector of the residuals which is assumed to be normally distributed with $\text{e}\sim \text{N }(0,\text{I}\sigma_{\text{e}}^{2})$.

Furthermore, the coefficient of genetic variation (CV_g_) was defined as the square root of the additive genetic variance divided by the mean of the trait:


$${\text{CV}}_{\text{g}}\%=\frac{\sigma_{\text{g}}}{\bar{\text{X}}}\times 100$$

#### Genomic prediction

The genomic predictions were performed for animals that had both phenotype and genotype records using the GBLUP method. GBLUP was applied using ASReml-SA v4.2 software [15] as follows:


$${\text{y}}_{\text{c}}=1\mu +\text{Zg}+\text{e}$$

where y_c_ is a vector of the trait of the observations for the trait adjusted for fixed effects; 1 is the vector of ones; μ is the overall mean; Z is the incidence matrix and g is the vector of the genomic values, following a normal distribution of $\text{g}\sim \text{N }(0,\text{G}\sigma_{\text{g}}^{2})$, where $\sigma_{\text{g}}^{2}$ is the additive genetic variance and G is the marker-based genomic relationship matrix. The genomic relationship matrix (G-matrix) was constructed using the genome-wide complex trait analysis (GCTA) v1.94.1 software package [16] which efficiently holds the genomic relationship between animals [17]. The following equation was used to create G-matrix based on marker allele frequencies:


$$\text{G}=\frac{\text{M}{\text{M}}^{\prime }}{2\;\Sigma_{\text{i}=1}^{\text{m}}{\text{p}}_{\text{i}}(1-{\text{p}}_{\text{i}})}$$

where, m is the total number of markers; p_i_ is the allelic frequency of ith marker and M is the matrix of centered genotypes.

#### Validation of models

We employed a repeated ten-fold cross-validation approach to assess genomic prediction accuracy. In brief, the entire dataset was randomly divided into ten equally sized groups for the 10-fold cross-validation. In this process, nine subsets, comprising 90% of the data were used as the training population, while the remaining tenth subset, representing 10% of the data, served as the validation sample. This experiment was repeated 100 times, and a 10-fold cross-validation procedure was applied in each iteration [18]. Consequently, a training-testing procedure was replicated 10 times to ensure that each animal in the dataset had an opportunity to be included in both the testing and reference groups. This cross-validation technique was designed to minimize sampling errors [19]. Prediction accuracy was evaluated by calculating the average correlation between the adjusted phenotypes of individuals in the validation dataset and their GEBV. The accuracy for each replicate was determined as the mean of the accuracies obtained from the ten-fold cross-validations in the 100 replicates.

#### Estimation of heritability of the traits

The heritability (h^2^) values for each body conformation traits were calculated in Holstein populations using the following formula:


$${\text{h}}^{2}=\frac{\sigma_{\text{a}}^{2}}{\sigma_{\text{a}}^{2}+\sigma_{\text{e}}^{2}}=\frac{\sigma_{\text{a}}^{2}}{\sigma_{\text{p}}^{2}}$$

where, $\sigma_{\text{a}}^{2}$ is the genetic variance, $\sigma_{\text{e}}^{2}$ is the residual variance, and $\sigma_{\text{p}}^{2}$ is the phenotypic variance.

## RESULTS AND DISCUSSION

### Summary statistics of the phenotypic data

The summary statistics for the phenotypic data of the Korean Holstein population’s 25 body conformation traits are presented in Table 2. The linear body conformation traits are divided into four main categories: body traits (including stature, height at front end, chest width, body depth, angularity, body condition score, and locomotion), rump traits (such as rump angle, rump width, and loin strength), feet and leg traits (comprising rear leg set, rear leg rear view, foot angle, heel depth, and bone quality), and udder traits (encompassing udder depth, udder texture, udder support, fore udder attachment, front teat placement, front teat length, rear udder height, rear udder width, and rear teat placement). The average scores for the body traits ranged from 4.39 to 6.81, while the average scores for the rump traits ranged from 4.58 to 5.53. In the studied population, the scores for feet and leg traits varied from 5.01 to 5.62. In contrast, the average scores for udder traits ranged from 4.30 to 6.88. Among these traits, the highest coefficient of variation (CV) of 32.18% was observed in rear udder width, while the lowest CV (3.67%) was observed for overall conformation score.

### Heritability of body conformation traits

The GBLUP model was utilized to estimate additive genetic variances and residual variances, which were then used to derive heritability (h^2^) for body conformation traits, as shown in Table 3. In the Korean Holstein population, h^2^ values for body traits, rump traits, feet and leg traits, and udder traits ranged from 0.10 to 0.50, 0.21 to 0.35, 0.13 to 0.29, and 0.05 to 0.46, respectively. Locomotion exhibited the highest h^2^ value of 0.50 among the body traits, while rump angle had the highest h^2^ value of 0.35 among the rump traits. The rear leg set showed the highest h^2^ value of 0.29 among the feet and leg traits, and udder texture had the highest h^2^ value of 0.46 among the udder traits. The standard errors of the h^2^ estimates were all ≤0.08. On average, rump traits displayed the highest h^2^ values (0.29), while the feet and leg traits exhibited the lowest estimates (0.21).

The h^2^ of body conformation traits in Korean Holstein cows falls predominantly within the moderate to low range, which is consistent with previous findings in Chinese Holsteins [20] and Holstein populations from other countries [3,21]. The h^2^ of body conformation traits varied across lactations, similar to observations in studies on Korean Holstein cows [22]. It is worth noting that the h^2^ of stature was slightly lower in Chinese Holsteins (0.37) [20] and Brazilian Holsteins (0.39) [21] populations compared to the studied Korean Holstein cattle. According to the national-level database, the heritability estimates for stature, chest width, body depth, angularity, body condition score, locomotion, rump angle, rump width, rear leg set, rear leg rear view, foot angle, udder depth, udder support, fore udder attachment, front teat placement, front teat length, rear udder height, rear teat placement, and overall conformation score were as follows in the Korean Holsteins population: 0.320, 0.156, 0.265, 0.120, 0.187, 0.031, 0.307, 0.169, 0.116, 0.079, 0.067, 0.334, 0.107, 0.132, 0.172, 0.212, 0.169, 0.091, and 0.155, respectively. For the US Holsteins, the corresponding values were 0.43, 0.28, 0.35, 0.31, 0.31, 0.17, 0.35, 0.25, 0.19, 0.11, 0.12, 0.30, 0.17, 0.22, 0.27, 0.28, 0.20, 0.18, and 0.31 [23]. Additionally, h^2^ estimates for certain body traits in Canadian Holsteins [24], such as stature, height at front end, and body depth, were comparatively higher than the estimates in the current study. On the contrary, h^2^ estimates for angularity, body condition score, and locomotion in Italian Holstein cattle [25] were lower than those observed in the current study. Previous studies [6,26] reported varying h^2^ estimates for angularity, ranging from 0.11 to 0.33, which aligns with our estimate. The h^2^ for body condition score was consistent with previous findings in Holstein cows ranged from 0.10 to 0.34 [6,26], and the h^2^ for locomotion was lower in previous findings, ranging from 0.06 to 0.11 [26]. However, the h^2^ for locomotion were higher compared to the value of 0.03 found in first-parity Czech Holsteins [6]. The discrepancies in h^2^ estimates for body traits can be attributed to factors such as the trait definition, measurement type, statistical model employed, and included effects [26].

The h^2^ values for rump angle and rump width in our study were found to be 0.35 and 0.32, respectively. Comparing with other studies, Chinese Holsteins [20] reported a h^2^ estimate of 0.26 for rump angle, while their estimate for rump width was lower at 0.07. In contrast, Czech Holsteins [27] reported higher h^2^ values for both rump angle (0.31) and rump width (0.35). Brazilian Holsteins [21] estimated the h^2^ for rump angle at 0.40 and for rump width at 0.31. Considering dual-purpose Chinese Simmental cattle, rump traits showed moderate h^2^ ranging from 0.15 to 0.34. On the other hand, in Canadian Holsteins [24], the h^2^ value of loin strength was found to be 0.20, which was similar to our findings.

The h^2^ estimates of feet and leg traits in Korean Holstein cows were found to be within the low to medium range, which is consistent with earlier studies. The rear leg set had the highest h^2^ estimate (0.29), while heel depth had the lowest h^2^ (0.13). Comparatively, for Canadian Holsteins [24], the h^2^ estimates for rear leg set, rear leg rear view, foot angle, heel depth, and bone quality traits were 0.04, 0.11, 0.08, 0.08, and 0.27, respectively. For Czech Holstein cattle [6], the corresponding values were 0.12, 0.09, 0.08, 0.08, and 0.24, and for Chinese Holsteins [20], the values were 0.06, 0.08, 0.06, 0.05, and 0.05, respectively. It is worth noting that the h^2^ estimates for bone quality in Canadian and Czech Holstein populations were higher than those observed in our study.

In our studied population, a wide range of h^2^ patterns was observed in udder traits, ranging from very low (0.05) to medium (0.46) values. Specifically, in Canadian Holsteins [24], the h^2^ values for udder depth, fore udder attachment, front teat placement, and rear teat placement were 0.41, 0.26, 0.29, and 0.30, respectively, which are higher compared to our study. However, concerning udder traits, front teat length demonstrated a similar h^2^ to the Canadian Holstein population, with a value of 0.29. On the other hand, in the case of Chinese Holsteins [20], the h^2^ values for udder depth, udder texture, fore udder attachment, front teat placement, front teat length, rear udder height, rear udder width, and rear teat placement were 0.15, 0.09, 0.15, 0.10, 0.05, 0.13, 0.13, and 0.20, respectively. In our studied Korean Holstein population, front teat placement and rear udder placement displayed extremely low h^2^, suggesting a significant influence of environmental conditions on these traits. This indicates that improving these traits through selection alone may be challenging due to the strong influence of environmental factors. The low h^2^ observed for these traits suggests limited potential for significant response to selection and highlights the contribution of nonadditive genetic and environmental factors in explaining the observed variation [28]. Discrepancies in h^2^ estimates can be attributed to factors such as population differences, scoring systems, estimation methods, sample sizes, measurement errors, and statistical models employed [21,29,30]. It is important to note that udder conformation traits, such as the shape, location, and strength of attachments, exhibit h^2^ and significantly impact a dairy cow’s milk production capacity, consequently influencing culling decisions [31]. Specifically, udder depth plays a crucial role in udder health, as it is associated with somatic cell count (SSC) [32]. Cows with lower udder depth tend to have higher SSC levels, which have a noticeable effect [33]. Our study reveals a range of h^2^ patterns for various udder traits in our studied population, with some values higher or similar to those observed in Canadian Holsteins and Chinese Holsteins. Understanding the heritability of these traits is essential for breeding programs and management strategies aimed at improving udder health and milk production capacity in dairy cows.

According to CV_g_, the results of the study also indicated comparatively lower levels of additive genetic variation for the examined traits. The significant additive genetic variation for body traits ranged from 4.67% to 16.46%, for rump traits it ranged from 8.65% to 13.26%, for feet and leg traits it ranged from 6.67% to 13.00%, and for udder traits it ranged from 2.37% to 17.94%. Notably, the highest level of additive genetic variation was observed in rear udder width, reaching 17.94%. The evolvability of a trait is influenced by its genetic variability, as suggested by Houle [34]. This genetic variability plays a crucial role in determining how easily traits can be modified through breeding efforts. In this context, it can be inferred that compared to other traits examined in the study, rear udder width has a higher predicted genetic gain when assessed on a standardized scale. This implies that there is a greater potential for targeted improvement of rear udder width through selective breeding, considering its higher level of genetic variation compared to the other studied traits.

### Evaluation of genomic estimated breeding value prediction accuracies

**Figure 1:**
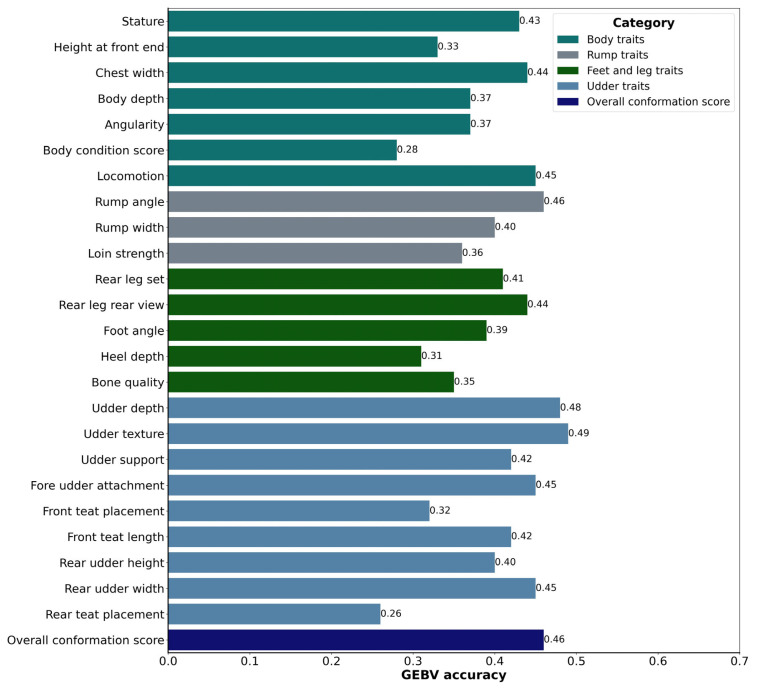
Comparison of genomic estimated breeding value (GEBV) accuracy for Korean Holstein body conformation traits in 10-fold cross-validation approach.

The accuracy of GEBVs is a critical measure in evaluating the reliability of genetic predictions for body conformation traits in Holstein cattle. In our study, we examined the GEBV accuracy for various body conformation traits to assess their predictive power and potential for genetic improvement which were presented in Figure 1. These traits are essential not only for comprehending the physical characteristics of the cows but also for making appropriate breeding decisions for improving the overall quality and productivity of the herd [35]. The GEBV accuracies for body traits in Holstein cattle ranged from 0.28 to 0.45. Specifically, in the case of stature, which denotes the height of the cow at her hips, it displayed an accuracy rating of 0.43, affirming the reliability of genomic predictions concerning this particular trait. Height at the front end, serving as an indicator of how the animal carries itself, estimated an accuracy score of 0.33. Meanwhile, chest width, a pivotal gauge of body width and conformation, was found at 0.44. Body depth, a critical trait for evaluating the overall physical structure of the cattle, attained an accuracy rating of 0.37. The desired characteristics for a cow include an angular, open, and well-sprung rib, accompanied by a wide chest and sufficient body depth, attributes that support the capacity for substantial milk production [35]. Angularity, which reflects the angular aspects of the body’s curves and lines, achieved a rating of 0.37. Additionally, the body condition score, signifying the amount of fat and muscle enveloping the cow’s bones, irrespective of body size, obtained an accuracy score of 0.28. Notably, locomotion, the measure of an animal’s ability to move effectively, achieved an impressive score of accuracy of 0.45.

On the other hand, rump traits had GEBV accuracies ranging from 0.36 to 0.46. Notably, rump angle, a pivotal attribute defining the curvature of the rump, achieved a substantial accuracy score of 0.46, underscoring its critical role in breeding programs. Meanwhile, rump width, a fundamental dimension of rump conformation, attained a commendable score of 0.40. In contrast, the assessment of loin strength, which gauges the vigor and stability of the loin region, yielded a less favorable accuracy rating of 0.36.

Similarly, accuracies for feet and leg traits, which are pivotal for the overall health and functionality of the cows, exhibited a range from 0.31 to 0.44. Rear leg set, an indicator of leg placement, scored at 0.41. Rear leg rear view, which assesses the rear leg structure from the rear view, achieved an accuracy of 0.44. Foot angle, a measure of the angle of the animal’s hooves, received an accuracy of 0.39. Heel depth, essential for evaluating hoof health, was recorded at 0.31. Bone quality, reflecting the strength and robustness of the cattle’s bones, achieved an accuracy of 0.35.

Furthermore, when examining udder traits that encompass the structural and qualitative aspects of the udder, a spectrum of accuracies emerged, ranging from 0.26 to 0.49. Udder depth, a crucial aspect of udder conformation, notably achieved a higher accuracy score of 0.48. Remarkably, udder texture, which assesses the texture of udder skin, yielded the highest accuracy rating at 0.49. Udder support, a vital attribute essential for optimizing milk production, demonstrated a commendable accuracy of 0.42. Fore udder attachment, a pivotal factor influencing udder health, registered a noteworthy accuracy score of 0.45. In contrast, front teat placement, serving as a gauge for teat positioning, received an accuracy score of 0.32. When assessing the length of front teats, front teat length achieved an average accuracy rating of 0.42. Rear udder height, reflecting the elevation of the rear udder, garnered an accuracy rating of 0.40. Equally pivotal, rear udder width, a fundamental parameter for evaluating udder conformation, achieved an impressive score of 0.45. Conversely, rear teat placement, which indicates the positioning of rear teats, yielded an accuracy rating of 0.26. Notably, udder texture exhibited a substantial accuracy of 0.49, which corresponds to the trait’s high heritability. The strong alignment observed between the high accuracy and heritability estimate for udder texture reflects the potential for accurate genetic predictions for this trait.

These accuracies provide valuable insights into the extent to which the GEBVs reflect the true genetic merit of the animals for specific traits. It is important to note that the accuracies we observed were generally lower, falling within the range recommended by BREEDPLAN, an Australia-based commercial company specializing in cattle evaluation. In BREEDPLAN, breeding values below 50% accuracy are considered preliminary and could undergo changes in the future with the inclusion of more direct performance information. On the other hand, values above 90% are highly reliable and less likely to significantly alter even with additional information. Breeding values falling between 50% to 90% accuracy represent varying degrees of reliability depending on the available information. The lower accuracies in the range can be attributed to factors such as the complex genetic architecture of these traits, limited available information on the animals, and the inherent challenges in accurately measuring and assessing these traits [36].

Over the past two decades, various statistical techniques have emerged for predicting GEBV. Notably, the genomic BLUP models and Bayesian variable selection or variable shrinkage models have gained widespread recognition and utilization. The idea of enhancing body conformation traits of Korean Holsteins by GS led to the estimation of GEBVs and their accuracy using GBLUP model, which presupposes a homogenous variance across SNPs and an equal contribution from each SNP to the overall variance [37]. Approximately a decade ago, Misztal et al [38] introduced a novel approach known as the single-step genomic BLUP method (ssGBLUP). This approach uses all available pedigree, genotypic, and phenotypic information, both from genotyped and non-genotyped individuals simultaneously. The use of ssGBLUP has been shown to significantly increase the accuracy of genomic prediction compared to methods that only utilize genotyped individuals. It’s important to note that maintaining accurate pedigree records can be a challenging and occasionally error-prone task. Despite these challenges, the GBLUP method remains a popular choice for practical genomic evaluations in dairy cattle. The widespread application of GBLUP within livestock species is primarily due to the polygenic nature observed in most traits [39]. Additionally, the GBLUP method is favored for its simplicity, lower computational requirements, and higher accuracy in contrast to the conventional pedigree-based BLUP (PBLUP) approach [37]. Other species have transitioned away from GBLUP to single-step methods, especially in dairy cattle, largely due to the cost and technical complexities associated with the latter. While GBLUP or SNPBLUP might act as an additional step to the PBLUP evaluation, they have been more straightforward to implement in dairy cattle compared to single-step methods, particularly when dealing with large datasets.

Among the body conformation traits, locomotion, rump angle, udder depth, udder texture, fore udder attachment, rear udder width, and overall conformation score exhibited the highest accuracies, with values of 0.0.45, 0.46, 0.48, 0.49, 0.45, 0.45, and 0.46, respectively. This suggests that these traits have a relatively stronger genetic basis and are more predictable through GEBV analysis. The higher accuracies observed for these traits indicate that genetic predictions for locomotion, rump angle, udder depth, udder texture, fore udder attachment, rear udder width, and overall conformation score can be relied upon with greater confidence in breeding decisions. It is important to acknowledge that the accuracies of GEBVs for body conformation traits are influenced by several factors, including heritability estimates, the size and quality of the reference population, and the availability of phenotypic and genomic data [40]. The accuracy of GEBVs can be further improved by increasing the size and diversity of the reference population, enhancing data quality, and employing advanced statistical methodologies.

## CONCLUSION

In conclusion, our study highlights the varying accuracies of GEBVs for different body conformation traits in Holstein cattle. While some traits demonstrated higher accuracies, indicating their suitability for selection and breeding purposes, others exhibited lower accuracies, suggesting the need for further research and refinement. Our results provide valuable guidance for breeders and stakeholders in the Korean Holstein industry. By leveraging the GEBV accuracy estimates, breeders can make informed decisions to optimize selective breeding strategies, ultimately leading to the enhancement of desired traits and the overall genetic progress of the population. Our study underscores the significance of genomic information in modern breeding practices and sets a foundation for future advancements in Holstein breeding programs. These findings contribute to our understanding of the genetic potential and predictability of body conformation traits in Holstein cattle and can inform breeding programs aimed at improving these traits in the future.

## Figures and Tables

**Table 1:** Description of linear type body conformation traits

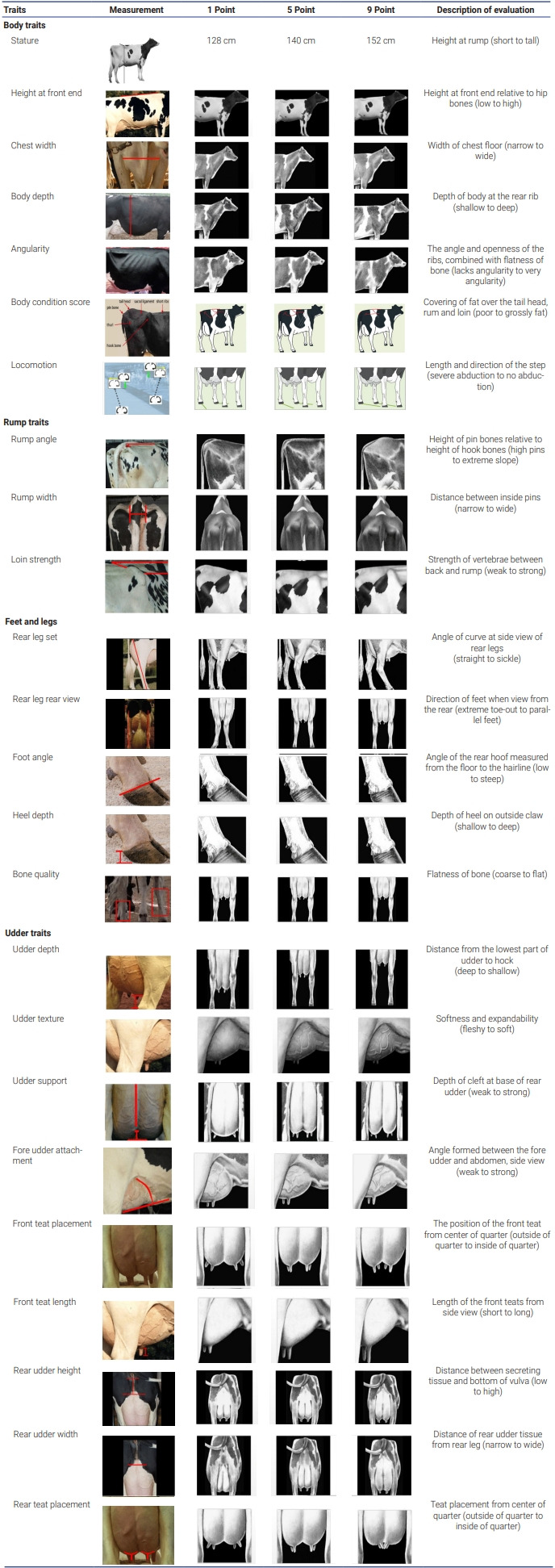

CRV [11]; Cho et al [12]; Holstein Association USA [13].

**Table 2:** Summary statistics for body conformation traits in the Korean Holstein population having both genotype and phenotype information

Traits	N	Mean	SD	CV%	Min	Max
Body traits						
Stature	732	6.81	1.28	18.84	1	9
Height at front end	713	4.88	0.60	12.23	3	8
Chest width	732	4.39	1.00	22.80	1	7
Body depth	732	4.75	1.00	21.21	1	8
Angularity	732	5.17	1.05	20.22	2	8
Body condition score	713	5.17	0.99	19.25	1	8
Locomotion	464	5.84	1.55	26.55	2	9
Rump traits						
Rump angle	732	4.75	1.05	22.14	1	9
Rump width	732	4.58	1.06	23.28	1	8
Loin strength	713	5.53	1.08	19.56	1	9
Feet and leg traits						
Rear leg set	732	5.01	0.99	19.73	2	9
Rear leg rear view	732	5.62	1.41	25.13	2	9
Foot angle	732	5.15	0.96	18.67	2	9
Heel depth/Hoof height	713	5.43	1.05	19.41	1	9
Bone quality	713	5.60	1.01	18.08	3	9
Udder traits						
Udder depth	732	6.40	1.16	18.18	3	9
Udder texture	713	5.44	1.36	24.99	2	9
Udder support	732	5.79	1.16	20.08	2	9
Fore udder attachment	732	5.67	1.17	20.67	1	9
Front teat placement	732	4.94	0.93	18.86	2	8
Front teat length	732	4.30	0.99	23.17	1	8
Rear udder height	732	6.88	1.18	17.22	1	9
Rear udder width	732	4.70	1.51	32.18	1	9
Rear teat placement	713	6.43	0.98	15.27	3	9
Overall conformation score	732	78.49	2.88	3.767	69	87

N, number of animals; SD, standard deviation; CV, coefficient of variation; Min, minimum; Max, maximum.

**Table 3:** Estimates of heritability, total phenotypic variance, additive genetic variance, residual variance, and coefficient of genetic variation for body conformation traits in Korean Holstein cows

Traits	h^2^	$\sigma_{\text{p}}^{2}$	$\sigma_{\text{g}}^{2}$	$\sigma_{\text{e}}^{2}$	CV_g_%
Body traits					
Stature	0.43 (0.08)	1.54 (0.09)	0.67 (0.14)	0.87 (0.11)	12.02
Height at front end	0.15 (0.07)	0.34 (0.02)	0.05 (0.02)	0.28 (0.02)	4.67
Chest width	0.25 (0.08)	0.87 (0.05)	0.22 (0.07)	0.65 (0.07)	10.70
Body depth	0.23 (0.06)	0.91 (0.05)	0.21 (0.07)	0.70 (0.07)	9.53
Angularity	0.22 (0.07)	1.02 (0.06)	0.22 (0.07)	0.80 (0.07)	9.05
Body condition score	0.10 (0.06)	0.91 (0.05)	0.10 (0.06)	0.82 (0.06)	5.98
Locomotion	0.50 (0.07)	1.87 (0.14)	0.92 (0.16)	0.94 (0.19)	16.46
Rump traits					
Rump angle	0.35 (0.08)	1.12 (0.06)	0.40 (0.09)	0.73 (0.09)	13.26
Rump width	0.32 (0.07)	1.11 (0.06)	0.36 (0.08)	0.75 (0.07)	13.10
Loin strength	0.21 (0.06)	1.11 (0.06)	0.23 (0.06)	0.88 (0.07)	8.65
Feet and leg traits					
Rear leg set	0.29 (0.08)	0.97 (0.05)	0.28 (0.06)	0.69 (0.07)	10.57
Rear leg rear view	0.28 (0.04)	1.91 (0.11)	0.53 (0.09)	1.37 (0.14)	13.00
Foot angle	0.17 (0.07)	0.81 (0.04)	0.13 (0.04)	0.67 (0.05)	7.13
Heel depth/Hoof height	0.13 (0.05)	1.01 (0.06)	0.13 (0.06)	0.88 (0.06)	6.67
Bone quality	0.18 (0.06)	0.94 (0.05)	0.17 (0.06)	0.77 (0.06)	7.26
Udder traits					
Udder depth	0.25 (0.06)	1.23 (0.07)	0.31 (0.08)	0.92 (0.09)	8.70
Udder texture	0.46 (0.05)	1.72 (0.10)	0.80 (0.08)	0.92 (0.12)	16.41
Udder support	0.19 (0.07)	1.24 (0.07)	0.24 (0.09)	1.00 (0.09)	8.47
Fore udder attachment	0.24 (0.07)	1.34 (0.07)	0.32 (0.09)	1.02 (0.09)	9.90
Front teat placement	0.05 (0.06)	0.84 (0.04)	0.04 (0.05)	0.79 (0.06)	4.23
Front teat length	0.35 (0.08)	0.97 (0.05)	0.33 (0.08)	0.63 (0.07)	13.44
Rear udder height	0.33 (0.05)	1.27 (0.07)	0.41 (0.08)	0.86 (0.07)	9.35
Rear udder width	0.32 (0.04)	2.24 (0.13)	0.71 (0.10)	1.53 (0.16)	17.94
Rear teat placement	0.09 (0.06)	0.94 (0.05)	0.08 (0.06)	0.86 (0.07)	4.40
Overall conformation score	0.45 (0.07)	7.73 (0.44)	3.45 (0.66)	4.28 (0.52)	2.37

N, number of animals; h^2^, heritability; $\sigma_{\text{p}}^{2}$, phenotypic variance; $\sigma_{\text{g}}^{2}$, genotypic variance; $\sigma_{\text{e}}^{2}$, residual variance; CV_g_%, coefficient of additive genetic variation. The number in parentheses are standard errors.
